# Anticipated Discrimination and Suicidal Behaviors Among Black Gay and Bisexual Male Adolescents: The Moderating Role of Developmental Assets

**DOI:** 10.21203/rs.3.rs-8435883/v1

**Published:** 2026-01-28

**Authors:** Donte T. Boyd, Arielle H. Sheftall

**Affiliations:** The Ohio State University; University of Rochester Medical Center

**Keywords:** suicidal behaviors, positive identity, social competencies, positive values, Black gay and bisexual male adolescents

## Abstract

**Background:**

Black gay and bisexual male adolescents (BGBMA) face disproportionately high rates of suicidal behaviors, shaped by intersecting racial and sexual minority stressors. Anticipated discrimination—a future-oriented minority stressor—may be an important yet understudied correlate of suicidality in this population. Guided by the Developmental Assets Framework, this study examined whether internal developmental assets (positive identity, positive values, social competencies) mitigate the association between anticipated discrimination and suicidal behaviors among BGBMA.

**Methods:**

Data were drawn from 387 BGBMA aged 14–17 years residing in three Midwestern U.S. cities who completed an anonymous online Qualtrics survey between December 2023 and January 2024. Suicidal behaviors were assessed using a self-report adaptation of the Columbia–Suicide Severity Rating Scale (C-SSRS). Anticipated discrimination was measured with a nine-item subscale of the Intersectional Discrimination Index, and internal developmental assets were assessed with validated scales of positive identity, positive values, and social competencies. Linear regression models tested main effects of anticipated discrimination and internal assets on suicidal behaviors, followed by moderation analyses examining interaction terms between anticipated discrimination and each asset.

**Results:**

Higher anticipated discrimination was significantly associated with greater suicidal behaviors (b = 0.46, *p* = .001). Positive identity (b = − 0.56, *p* < .001) and social competencies (b = − 0.78, *p* < .001) were each associated with lower suicidal behaviors. Two significant moderation effects emerged. Positive identity buffered the association between anticipated discrimination and suicidal behaviors (interaction b = − 0.23, *p* = .011); at high levels of positive identity, anticipated discrimination was no longer significantly associated with suicidal behaviors. In contrast, social competencies amplified the discrimination–suicidality association (interaction b = 0.49, *p* = .015), such that anticipated discrimination was most strongly related to suicidal behaviors at high levels of social competencies.

**Conclusions:**

Anticipated discrimination is a robust correlate of suicidal behaviors among BGBMA. Internal developmental assets do not function uniformly: positive identity appears protective, whereas higher social competencies may confer vulnerability under conditions of anticipated discrimination. Findings underscore the need for developmentally and culturally grounded suicide prevention strategies that both strengthen identity-related assets and address discrimination-related stress in Black gay and bisexual male adolescents.

## Introduction

Suicide remains a major public health concern, with more than 6,000 youth and young adults, ages 10 to 24 years, taking their lives in 2023.^1^ Epidemiological data consistently show males have higher suicide mortality rates than females, and one group that experiences an extremely high risk for suicide is the LGBTQ + group.^2–4^

LGBTQ + individuals are approximately three times more likely to consider suicide than their heterosexual counterparts and approximately five times more likely to attempt suicide, often using lethal means (e.g., a gun).^5^ According to the 2023 Youth Risk Behavior Surveillance Study, 40.6% of youth identifying as LGBTQ + seriously considered suicide, and one in five youth attempted suicide.^2^ These rates are alarmingly high and have persisted for LGBTQ + youth for approximately 20 years.^5^

Within the LGBTQ + community, Black gay and bisexual male adolescents (BGBMA) are at a particularly high risk for suicide and suicidal behaviors.^6^ Recent work by Boyd et al. draws on intersectionality and highlights how the convergence of racialized and sexual minority statuses exposes BGBMA to layered forms of racism, homophobia, and HIV-related stigma, which jointly elevate depression and suicidality.^7^ In a national sample of BGBMA, Boyd et al. demonstrated that suicidal ideation, planning, and attempts were highly concentrated among youth reporting low familial support and poor familial communication.^8^ These findings underscore the impact of interpersonal contexts on minority stress and its mental health consequences.

Complementary analyses of BGBMA (14–24 years) demonstrate that internal developmental assets (e.g., positive identity or coping skills) mitigate suicidal behaviors even when multiple minority stressors are present.^9^ These findings align with Mustanski and Liu’s longitudinal evidence that early exposure to minority stress escalates depressive symptoms and suicidality and correspond with Burton et al.’s work demonstrating that racism- and homophobia-related victimization exert distinct and deleterious effects on the mental health of Black sexual minority boys.^10–11^

Despite these contributions, most studies remain cross-sectional and emphasize distal outcomes (e.g., reported suicide attempts) rather than upstream processes such as youths anticipating and internalizing potential discrimination across social settings. Existing research demonstrates that anticipated discrimination consistently predicts elevated depression, anxiety, and suicidal ideation among racially and sexually minoritized populations.^15–16^ Studies of Black gay and bisexual men demonstrate how intersectional stigma—including racial, sexual, and HIV-related stigma—generates psychological distress and reduces engagement with preventive and mental health services.^17–19^ Research on internalized and anticipated stigma further indicates cumulative effects on depressive symptoms and suicidality, particularly among individuals facing socioeconomic marginalization.^20–21^

Anticipated discrimination is a critical, yet understudied, minority stressor that may help explain how intersectional stigma becomes “embodied” as depression, hopelessness, and suicidality among BGBMA. Despite the converging lines of evidence, the literature has not adequately examined how anticipated discrimination shapes mental health trajectories among Black sexual minority adolescents—a group simultaneously navigating identity development and heightened vulnerability to stigma-related stress. Emerging work documenting Black sexual minority youths’ experiences of racism and heterosexism^23^ signals the urgency of understanding these dynamics earlier in the life course, where cumulative exposure may influence long-term risk. Culturally grounded, developmentally tailored interventions fostering resilience, community connection, and mental health literacy are crucial to mitigating the adverse effects of anticipated discrimination during adolescence.^17;24–25^

### Developmental Assets Framework

The Developmental Assets Framework offers a strengths-based model for understanding how internal and external resources support healthy development, particularly during adolescence, characterized by increasing autonomy, identity exploration, and vulnerability to minority stress.^26–28^ According to the Developmental Assets Framework, youths thrive when they can access valuable internal assets (e.g., positive identity, coping skills, or decision-making competence) and external assets (e.g., supportive family relationships, caring adults, or safe community environments). Among BGBMA, these assets may function as vital buffers against the profound stressors associated with intersecting racial and sexual minority statuses.^26–30^ Recent empirical work among BGBMA demonstrates that internal assets, such as positive identity and coping skills, mitigate suicide risk even in contexts of racism, homophobia, and HIV-related stigma.^6–9^ External assets—including family support and affirming social networks—are similarly protective, reducing the likelihood of minority stress translating into depressive symptoms or suicidal behaviors.^12–13^ Further, research suggests that in the absence of these assets, psychological distress and suicide risk cluster together with broader health vulnerabilities.^7;14^ Collectively, the Developmental Assets Framework provides a robust model for conceptualizing how strength-based protective systems can counteract adversity among Black sexual minority youth. Despite this promise, the Developmental Assets Framework has not been fully applied to the specific, layered stress processes shaping mental health risks for Black sexual minority adolescents.

### Current Study

**Figure 1:**
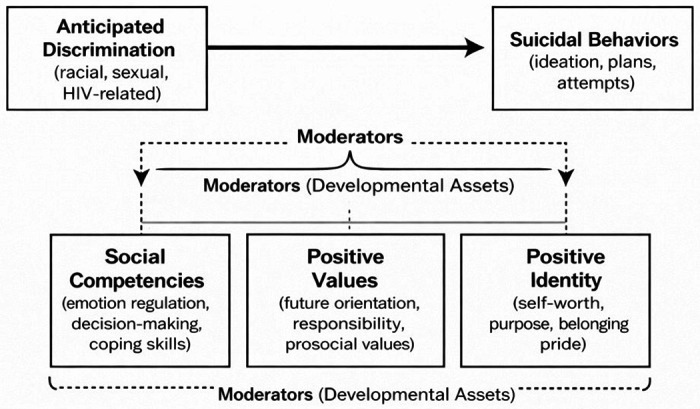
Conceptual Model of Anticipated Discrimination and Suicidal Behaviors Moderated by Developmental Assets **Note.** The model illustrates the hypothesized association between anticipated discrimination and suicidal behaviors among Black gay and bisexual male adolescents and young adults. Internal developmental assets (social competencies, positive values, and positive identity) are conceptualized as moderators that influence the strength of this association.

Grounded in the Developmental Assets Framework, the present study examines whether positive internal assets mitigate the association between anticipated discrimination and suicidal behaviors among BGBMA. Figure 1 presents the conceptual model guiding this study, illustrating the proposed associations between anticipated discrimination, developmental assets, and suicidal behaviors among BGBMA. The following research questions examined include:

1. How is anticipated discrimination associated with suicidal behaviors among BGBMA ages 14 to 17?
2. Do positive developmental assets (e.g., social competencies) moderate the relationship between anticipated discrimination and suicidal behaviors among BGBMA? If so, which assets are more protective?

We propose three hypotheses: (1) within BGBMA, higher levels of anticipated discrimination will be positively associated with suicidal behaviors; (2) higher levels of social competencies, positive values, and positive identity will be negatively associated with suicidal behaviors; and (3) social competencies, positive values, and positive identity will moderate the relationship between anticipated discrimination and suicidal behaviors.

## Methods

### Study Procedures and Recruitment

This study utilized an online survey to examine the influence of developmental assets on the sexual, physical, and mental health of BGBMA (14 to 17 years), as well as their suicidal behaviors. Participants resided in Midwestern cities of the USA.^9;31^

An anonymous link to the survey, generated by Qualtrics software, was embedded in a recruitment flyer.^9;31^ The research team promoted the survey through paid advertisements on social media platforms, including Facebook and X (formerly Twitter). Additionally, flyers were distributed through local community-based organizations and schools, with community health workers sharing them with eligible clients. Recruitment occurred between December 1, 2023, and January 31, 2024. All participants were required to provide assent and parental consent.

The study’s eligibility criteria included (1) self-identifying as Black/African American; (2) being between 14 and 24 years old; (3) residing in one of three Midwestern cities; (4) being assigned male at birth; (5) being fluent in English; (6) currently identifying as male; and (7) reporting sexual contact (oral, anal, or otherwise) with a male in the past year. For this study, responses from youth 14–17 years were only examined. Respondents who did not meet the inclusion criteria were excluded from the survey. To ensure all questions were answered, the Qualtrics forced-response option was used. Participants who completed the 20-minute survey and provided an email address received a $35 electronic Visa gift card.

The research team verified respondents’ IP addresses were located within the United States. Data integrity was further safeguarded by restricting each respondent to only one completion of the eligibility and survey questions. A speeding check was implemented to exclude participants whose completion time was less than one-third of the median survey duration. To ensure data quality, avoid duplicate submissions, and minimize the likelihood of bot activity, the survey employed Qualtrics survey protection measures. These measures included browser cookies placed upon survey submission, reCAPTCHA verification through preliminary questions requiring respondents to identify specific items in pictures or replicate a series of letters, and bot detection via a Qualtrics survey question that provided a reCAPTCHA score indicating the likelihood of a respondent being a bot.

Upon clicking the survey link, participants were presented with an informed consent form and a screening tool to determine their eligibility for the study. Eligible participants were asked a series of questions about demographics, developmental assets (e.g., academic engagement), and suicidal behaviors. Participants recruited through social media completed the survey on personal computers or smartphones while those recruited through community-based organizations used the organization’s devices.

### Measures

#### Suicidality

Suicidal behaviors were assessed using a self-report adaptation of the Columbia–Suicide Severity Rating Scale (C-SSRS), which has been psychometrically supported for self-report use in community and online samples.^32⊠33^ Although the C-SSRS was originally developed as a clinician-administered interview encompassing multiple domains of suicidal ideation and behavior, the present study focused specifically on the suicidal behaviors subscale, consistent with prior research examining behavioral indicators of suicide risk in nonclinical samples^9;11^. This subscale includes four dichotomous (Yes/No) items assessing the presence or absence of suicidal behaviors, including actual, interrupted, and aborted suicide attempts, as well as preparatory behaviors (e.g., writing a suicide note), assessed across the participant’s lifetime and within the past three months. Responses were summed to create a total suicidal behavior score, with possible values ranging from 0 to 4, where higher scores indicated endorsement of a greater number of suicidal behaviors. In addition, the number of suicide attempts was documented separately as a continuous variable. The subscale demonstrated strong internal consistency in this sample (Cronbach’s α = .87). To ensure participant safety, the survey concluded with a list of mental health and crisis resources, including the 988 Suicide and Crisis Lifeline, along with additional community-based supports.

#### Anticipated Discrimination

Anticipated discrimination was measured using a nine-item subscale drawn from a multidimensional discrimination battery assessing intersectional stigma experiences.^34^ This subscale captures participants’ expectations of experiencing unfair treatment in the future based on their social identities (e.g., race, gender, or sexual orientation). Participants rated the likelihood of each discriminatory event using a Likert-type scale ranging from 1 (very unlikely) to 5 (very likely), with higher scores reflecting greater anticipated discrimination. Example items include expectations of being disrespected, ignored, or stereotyped by others because of one’s identity. Item responses were averaged to create a composite score. Internal consistency reliability for the scale was excellent in the present sample (α = .92). Only the anticipated discrimination subscale was included in the current analyses, as it aligns with the study’s focus on the psychological burden of expected discriminatory treatment and its association with suicidal behaviors.

#### Developmental Assets

Developmental assets were assessed with three internal asset subscales derived from previously validated measures with established reliability and construct validity.^26–31^ These measures have been widely used in investigating youth well-being and thus provide a robust foundation for assessing developmental assets across diverse populations.

#### Internal Assets

*Positive identity* was assessed using a six-item scale with two subcategories: hope (three items) and self-esteem (three items). Each item was rated on a 5-point Likert scale, ranging from 1 (*Strongly disagree*) to 5 (*Strongly agree*), with higher scores indicating a more positive identity. The scale demonstrated adequate internal consistency (α = .71).

*Positive values* were assessed using a 10-item scale with four subscales that measured caring (three items), social justice (two items), integrity (two items), and responsibility (three items). Participants rated their level of agreement with each item on a 5-point Likert scale ranging from 1 (*Strongly disagree*) to 5 (*Strongly agree*). The combined 10-item scale demonstrated strong internal consistency (α = .89).

*Social competencies* were assessed via eight items across two domains: social-emotional skills (four items) and planning/decision-making skills (four items). Items were rated on a 5-point Likert scale ranging from 1 (*Strongly disagree*) to 5 (*Strongly agree*), with participants indicating their level of agreement with each item, including “Being good at making and keeping friends.” The scale demonstrated strong internal consistency (α = .88).

### Statistical Analysis Plan

All analyses were conducted using STATA 18 for descriptive and preliminary statistics and M-plus 8.11 for multivariate modeling. Prior to analyses, data were examined for univariate outliers, normality, and multicollinearity. Overall missingness across all study variables was minimal (< 2%) and no single variable exceeded 2% missing data. Given the low level of missingness and the cross-sectional design, missing data were handled using complete-case estimation, which is unlikely to bias parameter estimates.

Descriptive statistics were computed for all study variables, including anticipated discrimination, suicidal behaviors, and the developmental asset subscales. Bivariate correlations were examined to assess zero-order associations among constructs and to evaluate conceptual distinctions among asset indicators. Reliability statistics (Cronbach’s alpha) were calculated for all multi-item scales.

To examine the association between anticipated discrimination and suicidal behaviors, linear regression models were estimated, given that suicidal behaviors were operationalized as a continuous outcome. All models controlled for age, education, income, and relationship status. Model fit was evaluated using R^2^ values, and effect sizes were expressed as unstandardized regression coefficients (b) with corresponding standard errors (SE) and *p* values.

Moderation analyses tested whether developmental assets moderated the association between anticipated discrimination and suicidal behaviors. Mean-centered constituent variables were used to compute interaction terms (e.g., anticipated discrimination × positive identity) to reduce multicollinearity. Separate moderation models were estimated for each developmental asset, followed by a final model including all significant interaction terms. Significant interactions were probed using simple slopes analyses to estimate the association between anticipated discrimination and suicidal behaviors at low (− 1 SD), mean, and high (+ 1 SD) levels of the moderator. A developmental asset was interpreted as buffering when the association between anticipated discrimination and suicidal behaviors was weaker at higher levels of the asset. Multicollinearity diagnostics (variance inflation factors < 5) were used to confirm model stability. Results were compared for convergence and consistency across specifications.

## Results

### Descriptive Statistics and Correlations

Descriptive statistics and bivariate correlations for all study variables are presented in Table 1. Participants reported a mean suicidal behavior score of 7.06 (SD = 2.08) on the suicidal behaviors index (possible range: 0–12), indicating that, on average, youth endorsed multiple suicidal behaviors. Anticipated discrimination scores were relatively low (M = 3.00, SD = 2.76; range: 1–5). Developmental assets demonstrated moderate to high average levels, including positive identity (M = 3.31, SD = 0.80; range: 1–5), positive values (M = 3.88, SD = 0.85), and social competencies (M = 3.83, SD = 0.84).

As expected, anticipated discrimination was positively associated with suicidal behaviors, *r* (382) = .35, *p* < .01, indicating that higher levels of anticipated discrimination corresponded with greater suicidal behaviors. All developmental asset variables were negatively associated with suicidal behaviors, ranging from *r* = −.22 to −.40 (all *p* < .05), suggesting that youth with stronger internal assets reported fewer suicidal behaviors. Developmental assets were moderately intercorrelated: positive identity was significantly associated with positive values (*r* = .52, *p* < .01) and social competencies (*r* = .41, *p* < .01). Positive values and social competencies were also positively associated with each other (*r* = .44, *p* < .01). Anticipated discrimination showed small to moderate negative correlations with all internal assets, ranging from *r* = −.18 to *r* = −.28 (*p* < .05). No correlation exceeded .70, indicating that multicollinearity was unlikely to cause a concern for subsequent multivariate analyses.

### Main Effects

As hypothesized, anticipated discrimination was significantly and positively associated with suicidal behaviors across models (see Table 2). In the combined model, controlling for all moderators and their interaction terms, higher levels of anticipated discrimination predicted more suicidal behaviors (*b* = 0.46, *SE* = 0.13, *p* = .001). Consistent with Hypothesis 2, higher levels of positive identity (*b* = − 0.56, *SE* = 0.09, *p* < .001) and social competencies (*b* = − 0.78, *SE* = 0.17, *p* < .001) were each associated with fewer suicidal behaviors.

### Moderation Effects

#### Social Competencies

**Figure 2:**
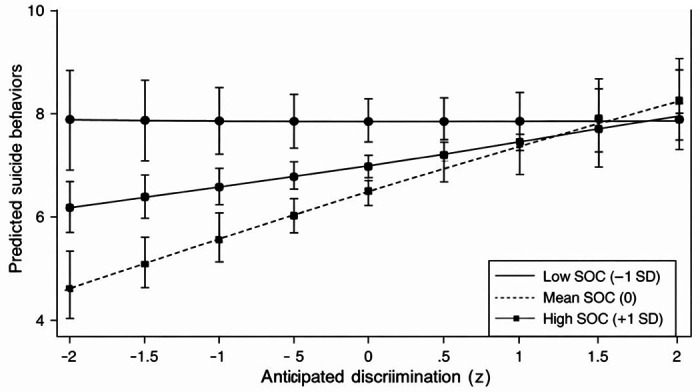
Interaction Between Anticipated Discrimination and Social Competencies Predicting Suicidal Behaviors **Note.** Predicted suicidal behavior scores are plotted as a function of anticipated discrimination (z-scored) at low (−1 SD), mean, and high (+1 SD) levels of social competencies. Solid, dashed, and dotted lines distinguish levels of social competencies in the black-and-white figure. Error bars represent 95% confidence intervals. Although social competencies were associated with lower suicidal behaviors overall, higher social competencies amplified the positive association between anticipated discrimination and suicidal behaviors.

The interaction between anticipated discrimination and social competencies was statistically significant (b = 0.49, SE = 0.20, p = .015). Simple slopes analyses indicated that the association between anticipated discrimination and suicidal behaviors was strongest among individuals with high levels of social competencies (+ 1 SD: b = 0.94, p < .001), moderate at the mean level (b = 0.46, p = .001), and nonsignificant among those with low levels (− 1 SD: b = − 0.03, p = .911; Fig. 2). This pattern suggests that, contrary to expectations, higher social competencies may heighten vulnerability to the psychological effects of anticipated discrimination, potentially reflecting increased social awareness, vigilance, or exposure to stigmatizing interactions.

#### Positive Values

The interaction between anticipated discrimination and positive values was not statistically significant in the full model (b = − 0.31, SE = 0.20, p = .136), indicating that positive values did not significantly moderate the association between anticipated discrimination and suicidal behaviors.

#### Positive Identity

**Figure 3:**
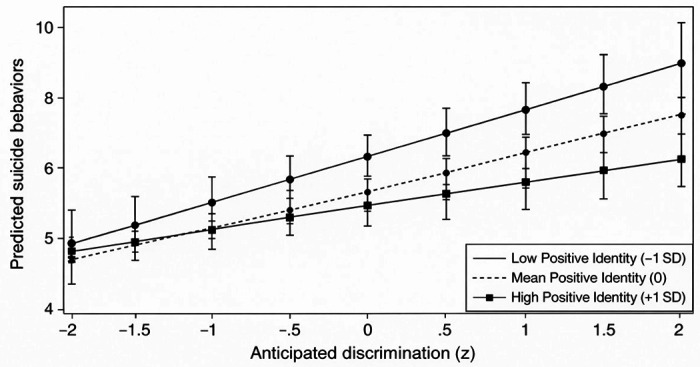
Interaction Between Anticipated Discrimination and Positive Identity Predicting Suicidal Behaviors **Note.** Predicted suicidal behavior scores are plotted as a function of anticipated discrimination (z-scored) at low (−1 SD), mean (0), and high (+1 SD) levels of positive identity. Solid, dashed, and dotted lines distinguish levels of positive identity in the black-and-white figure. Error bars represent 95% confidence intervals. The association between anticipated discrimination and suicidal behaviors was strongest at low levels of positive identity and attenuated at high levels, indicating a buffering effect.

Consistent with our hypothesis, the interaction between anticipated discrimination and positive identity was statistically significant (b = − 0.23, SE = 0.09, p = .011). Simple slopes analyses indicated that the association between anticipated discrimination and suicidal behaviors was strongest among individuals with low levels of positive identity (− 1 SD: b = 0.69, p < .001), moderate at the mean level (0 SD: b = 0.46, p = .001), and nonsignificant among those with high levels of positive identity (+ 1 SD: b = 0.22, p = .160; Fig. 3). This pattern indicates that a strong and coherent sense of identity may mitigate the psychological toll of anticipated discrimination on suicidal behaviors.

## Discussion

This study is among the first to examine the association between anticipated discrimination and suicidal behaviors among Black gay and bisexual male adolescents and to test whether internal developmental assets buffer (or exacerbate) this association. Consistent with minority stress theory and prior evidence documenting disproportionate suicide risk among LGBTQ + youth, anticipated discrimination emerged as a robust predictor of suicidal behaviors.^2–4^ This finding extends previous work demonstrating that racism, homophobia, and HIV-related stigma contribute to elevated psychological distress and suicidality among BGBMA.^6–7⊠11⊠17⊠19^ The present study adds to this literature by identifying future-oriented expectations of discrimination—a proximal minority stressor—as a meaningful risk factor during a developmental period characterized by identity exploration and heightened vulnerability to stigma-related stress.

The results partially supported our hypotheses. Higher anticipated discrimination was positively associated with suicidal behaviors, consistent with prior research demonstrating that discrimination heightens symptoms of depression, anxiety, and suicidality among racially and sexually minoritized populations.^15–16^ Two internal developmental assets—positive identity and social competencies—were significantly associated with lower suicidal behaviors, aligning with the Developmental Assets Framework, which posits that internal strengths, such as identity, coping skills, and decision-making competencies, promote resilience and psychological well-being.^28⊠26^ However, only positive identity demonstrated a buffering effect, weakening the association between anticipated discrimination and suicidal behaviors. This moderated effect provides empirical support that a coherent sense of self—particularly pride, hope, and self-esteem—may protect Black sexual minority adolescents from internalizing discrimination. These findings complement work showing that positive identity reduces depression and suicidality among sexual minority youth^10⊠30^ and contribute to emerging work demonstrating that internal assets mitigate adverse outcomes among BGBMA.^9^

Unexpectedly, social competencies amplified the association between anticipated discrimination and suicidal behaviors at higher levels. Although counterintuitive, this pattern may reflect heightened social vigilance or empathic attunement among youth who are more socially skilled, making them more sensitive to cues of rejection or anticipated mistreatment. Prior qualitative work among sexual minority men suggests that higher social awareness can increase emotional labor, stress appraisal, and sensitivity to discrimination.^24^ These youth may also have broader social exposure, increasing opportunities to encounter stigma. Alternatively, high social competencies may coexist with internalized pressures to “successfully manage” stigma, making discrimination feel more personally threatening. These interpretations warrant further investigation.

The nonsignificant moderation for positive values is notable. Although descriptive patterns aligned with a buffering hypothesis, positive values did not significantly attenuate the discrimination–suicidality link. This suggests that moral orientations (e.g., social justice, responsibility, or caring) may be less protective against discrimination-induced distress than assets tied to personal identity and social-emotional functioning. Given the mixed evidence regarding positive values in other populations, future research should examine how these assets operate in Black sexual minority adolescents’ day-to-day experiences, including whether certain values may activate stress responses or moral injury when confronted with identity-based unfairness.

### Theoretical Implications

The current study advances theory in several important ways. First, the findings extend minority stress theory by demonstrating that anticipated discrimination—a future-oriented expectation rather than an enacted experience—is strongly associated with suicidal behaviors among BGBMA. This supports contemporary conceptualizations of minority stress as cumulative, anticipatory, and developmentally embedded, with effects that accrue even before discriminatory events occur.^21⊠23^ The salience of anticipated discrimination during adolescence and young adulthood underscores how stigma-related vigilance may become internalized early in the life course, shaping emerging mental health trajectories.

Second, this study integrates the Developmental Assets Framework with an intersectionality-informed approach, illustrating that internal assets operate differently across stressor types and asset domains. This integration responds to long-standing calls to adapt strength-based developmental models for youth whose environments are marked by racism, heterosexism, and HIV-related stigma.^27⊠26⊠7^ The observed patterns—particularly the differential moderating roles of positive identity versus social competencies—highlight that developmental assets may not function uniformly across marginalized populations. Rather, their protective or exacerbating effects may depend on how specific assets interact with the unique stress ecologies of Black sexual minority adolescents.

Third, the significant buffering role of positive identity emphasizes the central importance of identity-related strengths for Black sexual minority youth, who must integrate racial and sexual identities in contexts where both are frequently stigmatized.^7⊠17^ This finding suggests that positive identity may be a core developmental mechanism through which youth resist the internalization of discriminatory expectations. In contrast, the lack of buffering effects for positive values and the unexpected amplification associated with social competencies point to the need for more nuanced theoretical models that account for the complex ways in which assets can shape stress reactivity and coping. Together, these contributions underscore the importance of theory that is culturally grounded, developmentally informed, and responsive to the intersecting forms of stigma experienced by BGBMA.

### Clinical Implications

Findings from this study highlight several important implications for clinical practice with BGBMA. First, youth who anticipate future discrimination exhibit significantly elevated suicidal behaviors, underscoring the need for clinicians to assess anticipated discrimination—not solely past victimization—during suicide risk evaluations. Incorporating questions about expectations of mistreatment in school, health care, community, and family settings may help identify youth whose stress exposure is not yet observable through enacted events but is nonetheless clinically significant.

Second, positive identity emerged as a robust protective factor, suggesting that interventions that affirm racial, sexual, and intersecting identities may play a critical role in mitigating the psychological consequences of discrimination-related stress. Strengths-based approaches that promote self-esteem, hope, and pride in one’s identities may be especially beneficial for BGBMA navigating stigmatizing environments.

Although social competencies were associated with fewer overall suicidal behaviors, they unexpectedly intensified the relationship between anticipated discrimination and suicidality at higher levels. This finding cautions clinicians against assuming that socially skilled or “high-functioning” youth are at low risk. Instead, youth with strong social competencies may benefit from additional support around managing stigma-related stress, emotional boundaries, and heightened social vigilance.

More broadly, suicide prevention efforts for Black sexual minority youth should integrate the Developmental Assets Framework with minority stress and intersectionality perspectives. Programs that strengthen internal assets must be paired with interventions that directly address racism, heterosexism, and HIV-related stigma across school, family, health care, and community contexts. Finally, family-focused and community-based approaches that promote family acceptance, open communication, and culturally grounded support remain essential. Given the central role of family and social environments in shaping the development of internal assets and exposure to stigma, enhancing these support systems is critical for reducing suicide risk among BGBMA.

### Limitations and Future Directions

Several limitations should be noted. First, the cross-sectional design limits causal inference, and future longitudinal work is needed to examine how anticipated discrimination shapes trajectories of suicidality over time. Second, the reliance on self-report data introduces potential biases, including social desirability and recall limitations. Third, although the sample represented three major Midwestern cities, findings may not generalize to youth in rural areas or other regions of the United States. Fourth, the anticipated discrimination measure, though reliable, was drawn from a broader discrimination battery and requires further validation for Black sexual minority adolescents specifically. Finally, future research should incorporate external developmental assets—such as family dynamics, school climate, and community connectedness—to expand the model into a more comprehensive ecological framework.

## Conclusion

This study underscores anticipated discrimination as an important and understudied predictor of suicidal behaviors among BGBMA. By integrating minority stress and the Developmental Assets Framework, the findings demonstrate that internal assets—particularly positive identity—play a critical role in protecting youth amid intersectional stigmas. These results highlight the urgent need for developmentally and culturally grounded suicide prevention programs that build internal strengths while addressing discrimination-related stress in the lives of Black gay and bisexual male adolescents.

## Figures and Tables

**Table 1:** *Descriptive Statistics and Correlations Among Key Study Variables* (N = *384)*

Variable	Mean	Standard deviation	1	2	3	4	5
1. Suicidal behaviors (C-SSRS)	7.06	2.08	–				
2. Anticipated discrimination	3.00	2.76	.35**	–			
3. Positive identity	3.31	0.80	−.40**	−.28**	–		
4. Positive values	3.88	0.85	−.33**	−.25**	.52**	–	
5. Social competencies	3.83	0.84	−.22**	−.18*	.41**	.44**	–

*Note*. Range = Suicidal behaviors (0–10); Anticipated discrimination (1–5); Positive identity (1–5); Positive values (1–5); Social competencies (1–5).

* *p* < .05.

*** *p* < .01.

**Table 2:** Main Effects of Anticipated Discrimination and Internal Assets on Suicidal Behaviors

Predictor	b	SE	t	p	β
Anticipated discrimination	0.53	0.07	7.05	< .001	0.27
Social competencies	−0.72	0.14	−5.12	< .001	−0.29
Positive values	0.10	0.13	0.72	.472	0.04
Positive identity	−0.57	0.10	−5.52	< .001	−0.22
Intercept	9.61	0.58	16.57	< .001	—
Model fit: R^2^ = 0.27; Adjusted R^2^ = 0.27; F(4, 527) = 49.69, p < .001; Root MSE = 1.69					

**Note.** b = unstandardized regression coefficient; SE = standard error; β = standardized coefficient.

Suicidal behaviors were modeled as a continuous outcome. All predictors were entered simultaneously. Higher scores indicate greater levels of each construct.

**Table 3:** Moderation of the Association Between Anticipated Discrimination and Suicidal Behaviors

Predictor	*b*	*SE*	*t*	*p*	95% CI [LL, UL]
Model 1: Anticipated Discrimination × Social Competencies					
Anticipated discrimination (AD)	0.46	0.13	3.44	< .001	[0.20, 0.72]
Social competencies	−0.78	0.17	−4.44	< .001	[− 1.12, − 0.43]
AD × Social competencies	0.49	0.20	2.44	.015	[0.09, 0.88]
Model 2: Anticipated Discrimination × Positive Values					
Anticipated discrimination	0.46	0.13	3.46	< .001	[0.20, 0.72]
Positive values	−0.48	0.10	−4.92	< .001	[− 0.68, − 0.29]
AD × Positive values	0.05	0.13	0.42	.675	[− 0.20, 0.30]
Model 3: Anticipated Discrimination × Positive Identity					
Anticipated discrimination	0.71	0.09	7.76	< .001	[0.53, 0.89]
Positive identity	−0.58	0.09	−6.27	< .001	[− 0.76, − 0.40]
AD × Positive identity	−0.21	0.09	−2.30	.022	[− 0.39, − 0.03]

Note. All predictors are standardized (*z*-scores). Each model tested one moderator at a time with robust *SE*s. The model adjusted for the following covariates: participant’s age, household income, homelessness

**Table 4:** Conditional Effects of Anticipated Discrimination on Suicidal Behaviors at Low, Mean, and High Levels of Each Moderator

Moderator	Moderator level	*b*	*SE*	*t*	*p*	95% CI [LL, UL]
Social competencies	−*1 SD*	−0.03	0.27	−0.11	.911	[− 0.55, 0.49]
	0 (*M*)	0.46	0.13	3.44	< .001	[0.20, 0.72]
	*+ 1 SD*	0.94	0.21	4.52	< .001	[0.53, 1.35]
Positive values	−*1 SD*	0.76	0.27	2.79	.006	[0.23, 1.30]
	0 (*M*)	0.46	0.13	3.46	< .001	[0.20, 0.72]
	*+ 1 SD*	0.15	0.21	0.74	.462	[− 0.26, 0.56]
Positive identity	−*1 SD*	0.69	0.16	4.27	< .001	[0.37, 1.01]
	0 (*M*)	0.46	0.13	3.47	< .001	[0.20, 0.72]
	+ 1 *SD*	0.22	0.16	1.41	.160	[− 0.09, 0.54]

Note. Conditional effects computed using margins in Stata. Low = − 1 *SD*; mean = sample mean; High = + 1 SD.

## Data Availability

Data that support the findings of this study are available on request under a license agreement. Written applications can be made to the author corresponding author.
